# Automated detection and quantification of breast cancer brain metastases in an animal model using democratized machine learning tools

**DOI:** 10.1038/s41598-019-53911-x

**Published:** 2019-11-22

**Authors:** Dina Sikpa, Jérémie P. Fouquet, Réjean Lebel, Phedias Diamandis, Maxime Richer, Martin Lepage

**Affiliations:** 1 Centre d’imagerie moléculaire de Sherbrooke, Département de médecine nucléaire et radiobiologie, Université de Sherbrooke, Sherbrooke, Québec, Canada; 2 Department of Laboratory Medicine and Pathobiology, University of Toronto, Toronto, Ontario Canada; 3 Département de Pathologie, Centre Hospitalier Universitaire de Sherbrooke, Québec, Canada

**Keywords:** Software, Metastasis, Machine learning, Cancer imaging

## Abstract

Advances in digital whole-slide imaging and machine learning (ML) provide new opportunities for automated examination and quantification of histopathological slides to support pathologists and biologists. However, implementation of ML tools often requires advanced skills in computer science that may not be immediately available in the traditional wet-lab environment. Here, we propose a simple and accessible workflow to automate detection and quantification of brain epithelial metastases on digitized histological slides. We leverage 100 Hematoxylin & Eosin (H&E)-stained whole slide images (WSIs) from 25 Balb/c mice with various level of brain metastatic tumor burden. A supervised training of the Trainable Weka Segmentation (TWS) from Fiji was achieved from annotated WSIs. Upon comparison with manually drawn regions, it is apparent that the algorithm learned to identify and segment cancer cell-specific nuclei and normal brain tissue. Our approach resulted in a robust and highly concordant correlation between automated metastases quantification of brain metastases and manual human assessment (R^2^ = 0.8783; P < 0.0001). This simple approach is amenable to other similar analyses, including that of human tissues. Widespread adoption of these tools aims to democratize ML and improve precision in traditionally qualitative tasks in histopathology-based research.

## Introduction

Microscopic analysis of hematoxylin and eosin (H&E)-stained slides prepared from tumor tissue remains the gold standard for clinical cancer assessment and diagnosis and a vital tool for cancer research^1,2^. Even in the research setting this often requires highly trained pathologists which are not always available. Performing this task accurately is crucial, especially when histology serves as ground truth in the treatment decision process. Similarly, in the laboratory, accurate quantification of histological sections is an important final step for validating hypotheses in *in vivo* animal models. Traditionally however, these have been done manually and in a relatively quantitative manner.

The advent of high-resolution whole slide imaging systems (or digital pathology) now allows the development and validation of computer-assisted tissue analysis methods, with the aim of automating some aspects of the process and of assisting pathologists and researchers in the detection, quantification, and classification of neoplastic disease burden^3–6^. However, the large amount of imaging data generated brings its challenges in terms of data storage and processing capability that are often beyond the scope of traditional biological research programs^7^. Specifically, machine learning (ML), “a subdomain of artificial intelligence”, enables computers to learn from a training dataset and extend their learned knowledge to subsequent cases for automated predictions^8^. ML opens up possibilities for automated and objective analysis of large datasets^9^. In biology, ML tools (e.g., deep learning, support vector machines and random forests) have found applications in fields such as proteomics^10,11^, genomics^12,13^ and radiomics^14–16^. Histopathological analysis has received significant attention in recent years due to emerging advances in ML tools for computer vision^17–21^. Despite these promises, many physicians and researchers, who stand to benefit from these technologies, do not have the computer science training or personnel to routinely implement these powerful technologies into their research program.

Consequently, a considerable effort has been made to develop image analysis platforms that depend on a relatively low level of technical expertise for application in digital pathology. Among the most popular are ImageJ^22^ and its distribution Fiji^23^, Icy^24^, Ilastik^25^ and CellProfiler^26,27^. Such open source software can be easily extended with plugins, scripts, pipelines or workflows. Based on these, researchers with software development skills can design more specialized and more customized analysis packages. However, they cannot handle the visualisation and processing of WSIs. OpenSlide^28^, Sedeen^29^ and QuPath^30^ offer an alternative to handle whole slide format. However, OpenSlide lacks an image analysis capability. Sedeen and QuPath both offer a comprehensive package (annotations, image analysis and automation) but Sedeen is still under development (the pathology image informatics platform, PIIP) and QuPath requires powerful computers with very high storing capacity and performance.

In this paper, we present a simple user-oriented approach for the automatic quantification of breast metastatic disease from histological mouse brain digital images. Metastases were implanted in Balb/c mice by intracardiac injection of the 4T1 murine mammary epithelial cancer cells which mimic stage IV human triple negative breast cancer^31,32^. The resulting brain tumor metastases were quantified using the trainable WEKA (Waikato Environment for Knowledge Analysis) Segmentation (TWS) plugin from Fiji (https://imagej.net/Trainable_Weka_Segmentation). This open source, user-friendly machine learning tool was designed to help carry out image segmentation using a supervised image classification approach^33^. Using the latter approach, a classifier is created from a training set of pixels manually attributed to different classes to reliably discriminate between classes and perform segmentation of a large number of images. The TWS plugin combines the image processing toolkit Fiji with machine learning algorithms from the data mining and machine learning toolkit WEKA^34^ to perform image segmentation based on pixel classification. WEKA contains a collection of tools and algorithms for data analysis and predictive modeling. Fiji, a distribution of Image J, is an open source image processing package providing a fast and easy access to powerful tools to explore and develop new image processing techniques^23^. Its ease of use and interactivity makes it attractive to scientists who need to perform advanced image analysis despite limited experience with programming. Thus, the paper serves as a proof-of-concept exercise to highlight to non-experts how this tool can be customized and implemented in the biological sciences. In this study, a training dataset was generated with representative and annotated tumor images. The algorithm was subsequently applied to the remainder of the dataset. Results obtained by automatic quantification were compared with manual segmentation.

## Methods

### Experimental data

Our dataset consists of 100 digitized H&E-stained brain sections from 25 Balb/c in which various levels of brain metastases were present. Briefly, Balb/c mice received an intracardiac injection of 4T1 breast cancer carcinoma cells (10^5^ cells in 100 µL PBS) into the left ventricle, which represents a model for haematogenous dissemination and metastatic invasion to the brain. Brain metastases can be detected as early as 5 days after cancer cells injection^35^. Eighteen days after intracardiac injection mice were sacrificed under deep anesthesia and brains collected for subsequent histological analysis and metastases quantification. Tissue slides were digitized using the Hamamatsu NanoZoomer 2.0-RS digital slide scanner (Hamamatsu Photonics, Hamamatsu City, Shizuoka, Japan) at a 40x magnification (i.e. 227 nm/pixel).

### Image preprocessing

**Figure 1:**
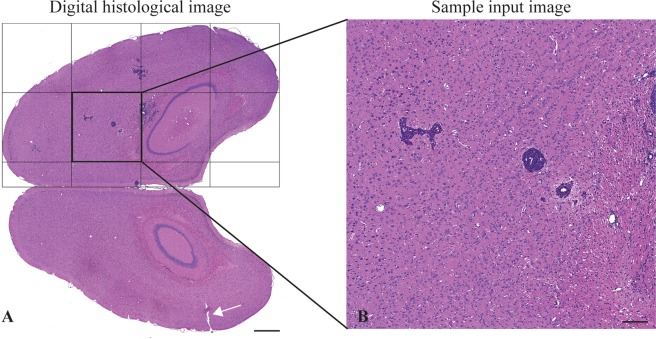
Image preprocessing. Representative H&E stained brain section showing histopathological features for breast cancer brain metastases. Cell nuclei and cytoplasm are stained in purple and pink, respectively. (**A**) The left brain hemisphere is identified by a small incision (white arrow - (magnification 1 × ; scale bar: 1 mm)). (**B**) Each hemisphere is automatically split in smaller images of at most 1024 × 1024 pixels in size (magnification 5 × ; scale bar: 200 µm).

Whole slide images were preprocessed because of their large size at full resolution. First, images were resized at a 5x magnification with the NanoZoomer NDP.view2 viewing software. Then, using MATLAB, brain hemispheres were extracted as left and right hemisphere (LH and RH, respectively). Each hemisphere was subdivided in smaller images (tiles) containing 1024 × 1024 pixels, creating a substack of images (8–15 tiles per hemisphere) as illustrated in Fig. 1. This strategy reduced data volume and allowed to process images in each substack as individual images to increase the training examples for supervised learning.

### Machine learning

The TWS plugin in Fiji (version 3.2.20) is an open source machine learning and data mining toolkit^33^ based on the Waikato Environment for Knowledge Analysis (Weka, University of Waikato, Hamilton, New Zealand).

**Figure 2:**
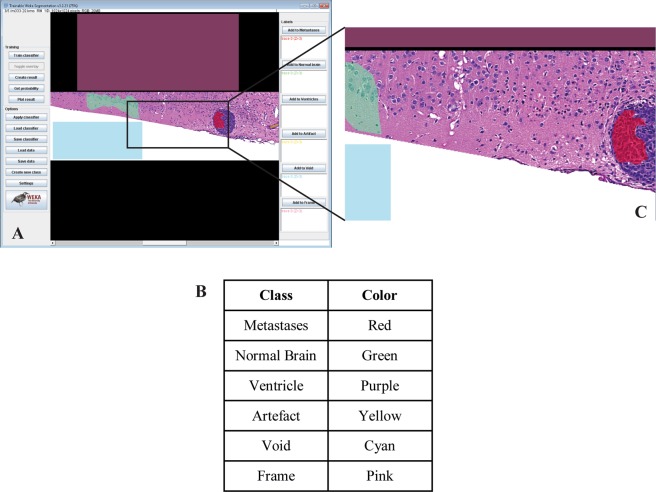
Image classification with supervised machine learning. The TWS graphical user interface (GUI) allows supervised training. (**A**) The classifier is trained with a set of representative images annotated with regions of interest (ROIs) allocated to the corresponding pre-defined classes listed in (**B**). In this example, ROIs are attributed to the Void (cyan), Normal brain (green), Frame (pink), Metastases (red) or Artefact (yellow) class. (**C**) Image magnification. Here, the manually drawn ROI belonging to the Metastases class includes cells nuclei and cytoplasm. For images smaller than 1024 × 1024, a black frame is created when images are loaded into the TWS GUI. Those pixels are attributed to the Frame (pink) class.

Six segmentation classes were created: Normal Brain, Metastases, Ventricles, Artefact, Void, and Frame. The Artefact class was added since brain slide images can present artefacts related to tissue sectioning (folds, chatter) and staining (precipitates). The Frame class was added to account for a black frame present for tiles smaller than 1024 × 1024 pixels when stack-loaded in the TWS plugin (Fig. 2).

We developed our application with the following features: Hessian, Laplacian, Sobel filter, Difference of Gaussians (edge detectors filters), Mean, Maximum, Variance, Median (pixel intensity and texture filters), Gaussian Blur, Anisotropic diffusion, Kuwahara (noise reduction filters) and Membrane projections (membrane detector)^33^. Default settings were kept for membrane thickness, membrane patch size, minimum sigma, and maximum sigma options (1, 19, 1.0 and 16.0, respectively).

The default TWS Random Forest (RF) classifier was selected with the following options: maxDepth = 20; numFeatures = 2; numTrees = 80. Classifier options were chosen considering a balance between segmentation performance and cost in processing time (due to image size). As shown previously^36^ and according to our own visual inspection, reducing the number of trees from 200 (default value) to 80 did not affect segmentation performance.

The classifier was trained with tiles from LH and RH to include intrinsic variability in brain structures and metastases morphology. Variability in the H&E stain leads to color variations (from pink to purple) on histological brain images and may compromise the automated segmentation. To overcome that variability, two classifiers were trained, one for slides that had brighter eosinophilic staining, and another for images that are darker. The classifier performance was evaluated by comparing segmentation to the visual evaluation of a board-certified pathologist. The out of bag error was maintained under 5%. The classifier was trained with 72 tiles and the training period lasted approximately 52 hours (Intel Core i7-4790 CPU @ 3.60 GHz, 4 cores, 32 Go RAM). Once trained, classifiers were saved and applied to the remaining data and the time needed to process a stack of 12 tiles (size of a subdivided hemisphere image) was 12–15 min. A script was created in Fiji to allow the user running a macro to select a folder (substack), run the TWS plugin, and then load and apply a classifier to all images contained in the stack. The results generated consisted in a stack of segmented images with index values corresponding to the segmented classes.

### Postprocessing of segmented images

Segmented images were refined with a final postprocessing step. False positive pixels (normal brain pixel misclassified as metastases) were present on some slides. We applied a filter in order to refine the classification and reduce misclassification of scattered pixels: metastases class objects with an area below five pixels were reattributed to the normal brain class. A threshold of five pixels was selected because misclassified pixels often appear as scattered groups of one to five pixels. Statistics for each class were automatically outputted to an Excel file.

### Manual segmentation

Brain metastases were manually outlined in all H&E stained brain slide digital images using NanoZoomer NDP.view2 viewing software free hand tool. Manual quantification served as a reference to evaluate the TWS metastases segmentation method. The relative area occupied by metastases in the RH and LH for each animal is the sum from all slides. All analyses were performed blinded to the experimental data.

### Statistical analysis

Statistical analysis was performed using GraphPad Prism 7.03 (GraphPad Software, Inc.). Pearson correlation analysis was used to asses association between automatic and manual methods. A value of P < 0.05 was considered significant.

## Results

### Image classification

In our model, metastatic lesions typically consist of cohesive, nest-forming neoplastic cells showing limited eosinophilic cytoplasm and bearing large, fairly round and hyperchromatic nuclei. An angiocentric tumor growth pattern was often noted.

**Figure 3:**
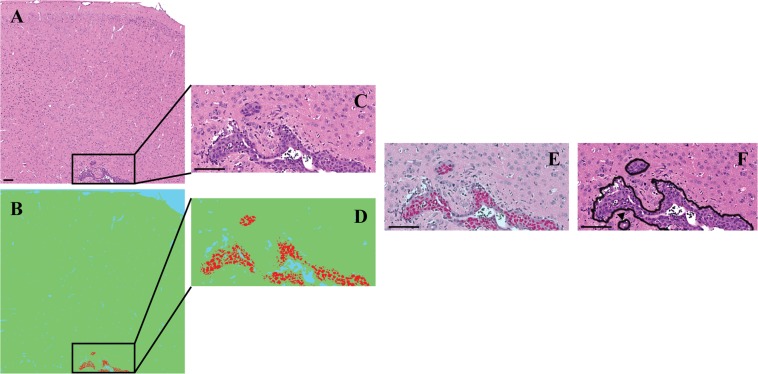
Example of automated and manual metastases segmentation. The trained classifier was applied to (**A**) new images in order to obtain (**B**) segmented images colored according to predefined classes. (**C**) Image magnification. (**D**) Corresponding region in the classified image showing accurate identification of metastases. (**E**) Image overlay confirming co-localisation. (**F**) The same metastases are detected using manual segmentation (black lines). A small metastasis is suspected below the larger one (black arrowhead). This small tumor is not detected in the automatically segmented image shown in panel D. Scale bar: 100 µm.

Figure 3A shows a metastatic tumor located at the grey and white matter junction, as it is often the case in human pathology. Figure 3B is the corresponding segmented image. In that image, blue, green, and red pixels correspond to voids (vessels lumen and background), normal brain tissue, and metastatic tumor cells, respectively. Tumor cells are accurately detected, as can be seen on Fig. 3B,D,E. However, metastatic volume is incompletely segmented. Figure 3E shows the segmented colour-coded areas overlaid on the original image. The classifier only identifies regions with denser and darker nuclei as part of the metastasis class. This suggests that nuclear features are preferentially exploited by the algorithm for tumor detection. This contrasts with the manual segmentation process which includes not only cell cytoplasm and nuclei but also intratumoral blood vessels (Fig. 3F).

We identified minor pixels misclassification related to the automatic segmentation. In Fig. 3F, one can see a small metastasis below the large tumor (black arrowhead), which was accurately delineated during the manual segmentation. The automatic classifier did not segment this lesion (Fig. 3D). This case illustrates the detection limits of the algorithm.

### Comparison of the TWS automated segmentation and manual segmentation

**Figure 4:**
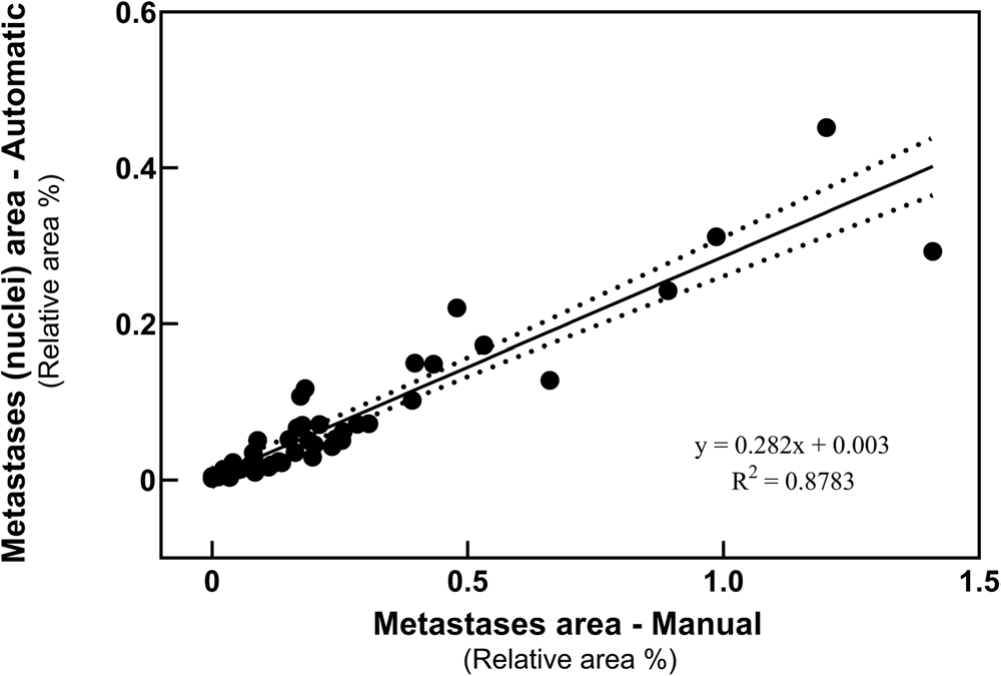
Comparison of metastases area derived from automated and manual segmentation. Pearson correlation analysis was performed to evaluate the quality of our automatic machine learning method against manual segmentation. The automatic quantification is positively and strongly correlated with the manual quantification of metastases (R = 0.8783; ****p < 0.0001).

Manual and automatic segmentation comparisons were performed to determine the classifier accuracy. To this end, we used histological brain slides from Balb/c mice with varying tumor burden. Slides included in the training dataset were excluded from the correlation analysis. As shown in Fig. 4, results from the two approaches are strongly correlated with a positive linear relationship. The Pearson’s correlation coefficient is greater than 0.8 and the *p*-value less than 0.0001. The areas measured manually are larger when compared with the automatic method.

## Discussion

This paper presents a simple and easy workflow to implement methodology for automatic brain metastases detection from histopathological image-based quantification using an open-access and readily available TWS plugin from Fiji. The results help validate that these tools can be readily trained to carry out accurate lesion segmentation of metastatic deposits. Indeed, a very strong correlation was found between automatic and manual methods.

The tumor cell/nucleus identification and segmentation problem is not new in digital pathology and has been well studied. The most common methods have been reviewed elsewhere^1,37,38^ and include thresholding (i.e., converting an intensity image into a binary image based on image pixel intensity using methods like Otsu or local thresholding), morphology, region growing (i.e., growing regions by connecting/classifying neighbouring pixels of similar intensity level) or watershed algorithms, active contour models and level sets (i.e., using splines to connect local maxima of the gradient image). Those image processing methods are improved by incorporating ML algorithms. For example, a threshold is used as an initial step for region identification (tumor vs normal region) then further processing such as feature extraction and classification is performed using more advanced ML algorithms^39^.

The automatic method was consistently more conservative when compared to the human annotator. This appears to be due to the algorithm’s focus on cell nuclei features as a major tumor recognition attribute, while humans circle the entire tumor area, which includes cell cytoplasm, nuclei and enclosed blood vessels. As mentioned previously, this can also be in small part attributed to the misclassification of some pixels when tumor cell nuclei are not intensely colored, making metastatic lesions difficult to distinguish even for a trained observer. Our study included only one observer; more observers would be required to assess intra-observer variability and to reduce user-induced bias. Despite these limitations, there was a strong overall correlation between human and machine estimates.

**Figure 5:**
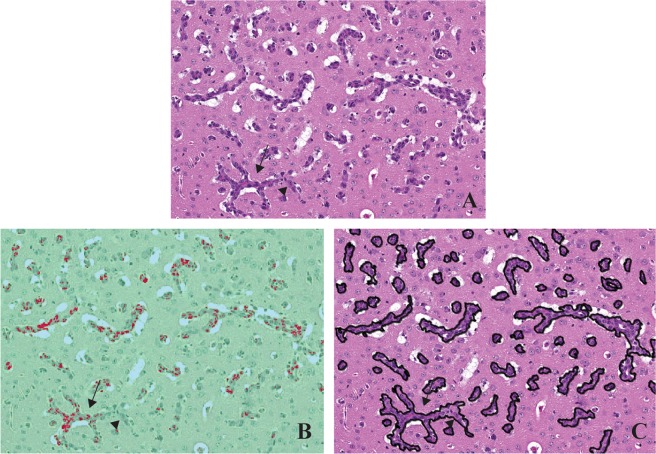
Detection limits of the automated method in cases with unusual tumor invasion patterns. Panel (**A**) shows a metastatic lesion with an unusually strong angiocentric pattern of invasion. In this setting, the automated classifier (**B**) underestimates tumor volume when compared to manual segmentation (**C**). Arrows point out focal detection variability: metastasis-brain parenchyma boundaries are not clearly delineated (arrowhead).

Metastatic histological features, including nuclear features and staining, cytoplasm, vacuolation (vessel lumen, oedema) and metastasis-brain parenchyma interface demarcation vary between tissues slices and within a single slice. As shown in Fig. 5, this can lead to atypical tumor patterns and impact on the performance of manual and automatic segmentation, thus explaining pixel misclassification in the former and erroneous tumor identification and delineation in the latter.

In our model, some tumors displayed an infiltrating pattern, which is somewhat less typical for epithelial secondary tumors. This invasive pattern was not consistently detected by the algorithm. Some tumors also showed a significant inflammatory component however, tumor infiltrating immune cells were not systematically identified by the algorithm (data not shown). Training data must include more of those specific cases. Validation of an algorithm trained with immunohistochemically stained slides for keratin to detect tumor could help in resolving quantification issues in this setting as it would reveal specifically tumor location.

The ML algorithm was trained with a set of images reflecting the heterogeneity in our data. Increasing the amount of training data, especially including more atypical tumor cases, might increase the automatic segmentation efficiency. It is possible that more advanced unsupervised ML algorithms (e.g., deep learning) could increase performance in such cases. Other methods for image segmentation exist. On one hand, we implemented simpler methods (such as thresholding or region growing) in exploratory studies leading to the current work. These suffered from important limitations and such an unfair comparison would not make a convincing or interesting case for our method. On the other hand, other advanced algorithms are often more complex, require specialized software and/or computer hardware (e.g. GPUs) larger datasets, and are not as user-friendly as the TWS software used in this study, or are not already available through open-source and free software (such as ImageJ in the case of TWS). Optimizing a full set of methods to make a fair and thorough comparison would require in-depth knowledge and comprehensive optimization of each method.

Our aim was instead to develop an image analysis approach that met the usability criteria destined for a broad community of users^40,41^:

- User-friendly: our approach is usable by non-experts; tutorials and data to reproduce our results are available through a public repository (see Supplementary information).
- Developer-friendly: the scripts and licensing are open source.
- Interoperable: Fiji was developed to facilitate interactions between imaging platform. For example, it is possible to run a Fiji plugin from Matlab, CellProfiler or Icy.
- Modular: our pipelines can serve as basis for future work, new functionality can be added easily. In fact, Fiji is a pioneer in extensibility.
- Validated: our approach has been tested with a heterogeneous dataset (staining, tumor morphology) and can be validated by future users with their own and/or our data.

In our approach, the final segmentation was highly impacted by classifier performance, which in turn is mostly influenced by the quality and reproducibility of the training data and feature selection. The training data must include all the aspects and variations of the structures to be segmented. Two classifiers were trained to account for variations in color staining. A uniform preprocessing with a step of color stain normalisation might have allowed us to use a single classifier.

Cancer cell nuclear features were the major determinant selected by classifiers models for metastases segmentation. Detection of the cell nucleus is relevant since their morphologic patterns change between cell-type, cancer type and cancer grade, and are key diagnostic features in cancer. Those patterns include shape, density, area (nucleus/cytoplasm ratio), intranuclear inclusion, changes in chromatin and mitotic count, and are markers of tumor malignancy. Nuclear grading has been shown to have a prognosis value in breast cancer and renal cell carcinoma^42–44^. Thus, our method could be easily adapted to other settings such as breast biopsies since the relevance of nuclear features in grading primary breast cancer is well established (Nottingham histologic score)^45,46^.

We conclude that our simple and user-friendly machine learning approach allows for the automatic detection of small and large groups of metastatic breast cancer cells on digital histological images. This can be very useful in reducing image analysis time both in preclinical and clinical research settings. While further development is possible, such tool could enable a faster and more accurate diagnostic prediction from tumor biopsies and consequently decrease the time required in patient management.

## Supplementary information

Supplementary Information

## Data Availability

Datasets are available for review from the corresponding author upon request.

## Code availability

A GitLab repository (weka4metastases), with scripts developed (WSI_2_Tiles.ijm, TWS_metastases.ijm) for this work and datasets to reproduce results presented in this paper, is available for revision at https://gitlab.com/lepage-mri-group/weka4metastases. You can download material from this repository and detailed instructions can be found at https://gitlab.com/lepage-mri-group/weka4metastases/blob/master/Readme.md.
