# Supplementary material for: Automated detection and quantification of breast cancer brain metastases in an animal model using democratized machine learning tools
Source: Sci Rep. 2019 Nov 22;9:17333. doi: 10.1038/s41598-019-53911-x (PMC6874643; doi:10.1038/s41598-019-53911-x)
Supplement: Supplementary file 1 — Supplementary Information [file 41598_2019_53911_MOESM1_ESM.docx]

**Automated detection and quantification of breast cancer brain metastases in an animal model using democratized machine learning tools**

**Author list and affiliations**

Dina Sikpa^1^, Jérémie P. Fouquet^1^, Réjean Lebel^1^, Phedias Diamandis^2^, Maxime Richer^3^, Martin Lepage^1*^

^1^ Centre d’imagerie moléculaire de Sherbrooke, Département de Médecine nucléaire et radiobiologie, Université de Sherbrooke, Sherbrooke, Québec, Canada.

^2^ Department of Laboratory Medicine and Pathobiology, University of Toronto, Toronto, Ontario

^3^ Département de Pathologie, Centre Hospitalier Universitaire de Sherbrooke, Québec, Canada

**Correspondence to:** Professor Martin Lepage

Département de médecine nucléaire et radiobiologie,

FMSS, Université de Sherbrooke

3001 12ème avenue Nord

Sherbrooke, Québec, Canada, J1H 5N4

E-mail: Martin.Lepage@USherbrooke.ca

**Supplementary information**

**Code availability:** A GitLab repository (weka4metastases), with scripts developed (WSI_2_Tiles.ijm, TWS_metastases.ijm) for this work and datasets to reproduce results presented in this paper, is available for revision at <https://gitlab.com/lepage-mri-group/weka4metastases>. You can download material from this repository and detailed instructions can be found at <https://gitlab.com/lepage-mri-group/weka4metastases/blob/master/Readme.md>.

**Supplementary video S1:** Movie tutorial demonstrating how to use the program to divide whole slide images into tiles (WSI_2_Tiles.ijm).

**Supplementary video S2:** Movie tutorial showing how to use the program for automatic metastases segmentation (TWS_metastases.ijm).
